# A Cross-Sectional, Exploratory Study on the Impact of Night Shift Work on Midwives’ Reproductive and Sexual Health

**DOI:** 10.3390/ijerph19138082

**Published:** 2022-07-01

**Authors:** Joanna Moćkun-Pietrzak, Aleksandra Gaworska-Krzemińska, Anna Michalik

**Affiliations:** 1 Department of Obstetric and Gynecological Nursing, Medical University of Gdansk, M. Sklodowskiej-Curie Street 3a, 80-211 Gdansk, Poland; joanna.mockun@gumed.edu.pl (J.M.-P.); aniamichalik@gumed.edu.pl (A.M.); 2 Department of Nursing Management, Medical University of Gdansk, M. Sklodowskiej-Curie Street 3a, 80-211 Gdansk, Poland

**Keywords:** reproductive health, shiftwork, night work, sexual health, midwife

## Abstract

Background: Shift work is the basis for health care system functioning. The non-standard schedules enforce abrupt changes in the timing of sleep and light-dark exposure. It can contribute to the increased risk of various medical conditions, including reproductive and sexual health issues. The purpose of the study was to assess the impact of shift work with night shifts on midwives’ reproductive and sexual health. Methods: This cross-sectional, exploratory study included 520 midwives. A descriptive questionnaire was distributed in person (414) and online (106) from July 2019 to May 2020. We used the Female Sexual Function Index (PL-FSFI) standardized questionnaire and proprietary research tools (applicable to demographic and social data and reproductive health). All statistical calculations were performed with the IBM SPSS 23 statistical package. Results: Shift work affects midwives’ reproductive and sexual health. Midwives working night shifts are more likely to experience reproductive problems and sexual dysfunctions. The most pronounced differences are observed in the experience of infertility and the number of miscarriages. PL-FSFI results clearly showed the adverse impact of working shifts including night shifts on functioning in various dimensions of sexual health. Conclusion: Shift work negatively affects reproductive and sexual health and causes work-life conflict experience. It is necessary to develop procedures that minimize shift rotation and implement work schedules that allow for recuperation or rest and ensure proper family and social life.

## 1. Introduction

According to Directive 2003/88/EC of the European Parliament and of the Council of 4 November 2003 concerning certain aspects of the organization of working time, shift work is any form of work organization in which individuals in the same positions work according to a specific schedule (including consecutive work), entailing the requirement to perform work at different times of the day and night. A shift worker is a person whose work schedule includes shift work [1].

In health care systems, shift work is a necessity in order to ensure the continuity and quality of services. Midwives ensure continuity in perinatal and gynecological care and community health. Most Polish midwives are employed in hospitals, which is associated with an irregular work schedule, including working nights and public holidays [2]. The International Council of Nurses proposed in 2007 that shift work was necessary in the nursing profession; however, there is also significant concern about the negative impact of working shifts on nurses’ physical and mental health and their ability to provide high-quality patient care [3].

The impact of shift work on workers’ physical and mental health has been studied for many years, and the results of reports indicate how vast and detrimental the consequences of this type of work can be. Changes in circadian rhythm are directly associated with disorders and may be a risk factor for many diseases. Among the most commonly described ones are sleep disorders, also known as the Shift Work Syndrome (SWS). Reports also indicate cardiometabolic disorders, including weight gain, type 2 diabetes, high blood pressure, atrial fibrillation, coronary heart disease, and stroke [3,4,5,6,7,8,9,10]. Night shift work can be a risk factor for breast cancer [6,9,11,12]. In addition, shift workers experience work-life conflicts, chronic fatigue, higher risk of depression and anxiety disorders [5,13,14]. Noticeably scarce reports focus on the impact of shift work on women’s reproductive health and sexuality. Existing evidence suggests that impaired circadian rhythm may be associated with a range of reproductive problems and sexual dysfunction: increased risk of irregular menstrual cycles, endometriosis, infertility, miscarriage or premature birth, low birth weight of a newborn, and reduced breastfeeding frequency [11,15,16,17,18,19]. It is noteworthy that among Polish nurses and midwives, there is a shorter life expectancy than in the average Polish woman for reasons that are yet to be explained [2]. Little is known about the impact of shift work on midwives’ reproductive and sexual health. Most of the research focuses on the professional group of nurses. The professional competence of midwives includes, among others, the education of women and the monitoring of reproductive health and sexuality parameters. Midwives have specific theoretical and practical knowledge in these areas. In addition, a larger scale of disorders in this area of life may be associated with a lower quality of life and correlate with a higher degree of professional burnout in this professional group. Therefore, it seems very important to examine midwives and provide feedback on the correlation between shift work and reproductive health, as they will be able to share their theoretical knowledge and their own experiences in this area while fulfilling their educational role among women. Midwives working at hospitals in Poland have limited ability to work only day shifts. Daytime care is performed by midwives working as hospital managers, surgical midwives, and midwives working in clinics, fertility clinics, and other places that do not provide night care. Midwives working at hospitals work in shifts, 7:00 a.m.–7:00 p.m. and 7:00 p.m.–7:00 a.m.

This study aimed to assess the impact of shift work including night shifts on midwives’ reproductive health and satisfaction with sexual life.

## 2. Methods

### 2.1. Study Design and Setting

This study was performed from July 2019 to May 2020 and included midwives employed in the Pomeranian Voivodeship (geographic region similar to a province or state) in the northern part of Poland; the Pomeranian Voivodeship has 2 millions 324,000 citizens).

A diagnostic survey was used as the research method. The research tool kit consisted of three elements:

The Female Sexual Function Index (FSFI) (PL-FSFI) translated into Polish [20,21], that consists of 19 items, and its goal is a multifaceted self-assessment of sexual functioning in the last four weeks. This tool allows for differentiating sexual dysfunctions in the areas of desire, arousal, orgasm, and sexual satisfaction. The cutoff point, which indicates sexual dysfunction in women, is ≤27.5 points. The use of the Polish version PL-FSFI of the sheet received regulatory approval.

The authors developed an original questionnaire. It contained questions regarding sociodemographic data (age, education, place of residence, state, civil status, financial situation, seniority, seniority in shift work, number of night shifts per month).

Reproductive health—contained original questions based on the review of the literature in this area (regularity of the cycle, length of the menstrual cycle, number of pregnancies, procreation problems, number of miscarriages, contraception used).

Researchers contacted midwives in person in their workplaces to distribute questionnaires. Each respondent was informed about the aim of the study and the planned method for the resulting publication. Each subject participated in the study voluntarily and provided informed consent. The completed questionnaires were returned to a sealed box later collected by the researchers or sent back by mail in the return envelope provided. Each participant was asked to fill in all the scales.

On 11 March 2020, the WHO assessed the coronavirus disease 2019 (COVID-19) outbreak as a pandemic; therefore, we opted to perform this study using the CAWI (Computer-Assisted Web Interview) method. The questionnaire was made available on Polish blogs dedicated to midwives on relevant social media sites. The link to this study was also distributed via online support groups dedicated to midwives. The questionnaire was made available with a link to an eligibility screener. Once a respondent exited a tool, they were unable to reopen it. Additional inclusion criteria were used here: only midwives from the Pomorskie Voivodeship were included. In total, 414 in-person and 106 online surveys were collected.

The inclusion criteria were met by 520 professionally active midwives working in the Pomeranian Voivodeship, which constitutes 25.68% of all registered midwives in this voivodeship [2]. The following exclusion criteria were applied: male gender, length of service below six months, and workplace outside the surveyed area. According to the guidelines for reliable factor analysis [22], the sample size was considered to be “fair”.

The protocol for the study was approved by the Independent Bioethics Committee for Scientific Research at the Medical University of Gdańsk (NKBBN/317/2019). Consent was also given by the management of the medical facilities where the study was conducted.

### 2.2. Statistics

All statistical calculations were performed with the IBM SPSS 23 statistical package and Microsoft Excel 2016 spreadsheet. The qualitative type variables were presented with the use of the number and percentage values, while the quantitative variable was characterized with the use of the arithmetic mean and the standard deviation. The significance of differences between more than two groups was assessed in the Kruskal–Wallis test (in the case of significant differences, Bonferroni post hoc tests were used and the Student’s *t*-test—between the two groups). A correlation analysis with the Spearman correlation coefficients was used to determine the association between force and direction between variables. For all the calculations, the significance level was set at *p* ≤ 0.05.

## 3. Results

### Demographic Characteristics

The study group consisted of 520 midwives (*n* = 416 midwives working shifts including night shifts and *n* = 104 midwives working only days, which reflects the actual proportion of midwives employed in both systems). The largest group of respondents (33.7%) was in the age group between 22 and 30 years old, and the least numerous (19%) in the group between 31 and 36 years old. The largest group of respondents was city inhabitants (87.7%) and had a university degree (Master of Science in Midwifery) (45.6%). The majority of the study subjects were married (61.7%), and 15.6% were cohabitating. The largest group among the surveyed midwives (39.6%) were subjects with over 16 years of experience, and the smallest (11%) one—with 11–15 years of experience. 28.3% of respondents had worked shifts for over 15 years, and 20% (*n* = 104) had never worked this kind of schedule. In the night shift group, the largest number of respondents declared that they had an average of 4–6 night shifts per month. Detailed demographic characteristics are presented in Table 1.

Midwives working shifts with night shifts were mostly 22–30 years old (X2(3) = 29.37; *p* = 0.000) and had 6 months–5 years or 5 years–10 years of professional experience (X2(3) = 29.44; *p* = 0.000). Shift workers were most frequently single or divorced (X2(3) = 13.17; *p* = 0.004) rather than married/ cohabitating or widowed. Other differences in demographic characteristics (level of education, place of residence urban/rural) weren’t significant.

The respondents were asked to subjectively assess their reproductive health and sexual performance using a standardized questionnaire: Female Sexual Function Index (PL-FSFI).

Table 2 presents data on reproductive health among individuals working shifts including night shifts and working only during the day. The data include the menstrual cycle length, obstetric history, and reported reproductive problems.

In the declarative assessment of reproductive health, there are differences between people working shifts including night shifts, and those working only during the day. The most notable differences indicating the detriment of shift workers are visible in reproductive problems, extended menstrual cycles, and the number of miscarriages (Table 2).

Additionally, we made analyses regarding menstrual cycle length and declared reproductive issues and miscarriages. They showed that statistically the length of menstrual cycle from 26 to 30 days most often occurs in women who work 4–6 and 7–9 night shifts per month, whereas the number of night shifts per month does not affect the regularity of the cycle. However, it has been demonstrated that reproductive problems were most often reported by women who have 4–6 and 7–9 night shifts per month, while the number of night shifts per month does not affect the number of miscarriages.

PL-FSFI results clearly showed the adverse impact of working shifts including night shifts on respondents’ functioning in various areas of sexual activity (Table 3). In each of the surveyed domains, respondents working shifts including nights achieved lower results. The result in the pain domain is read in reverse, i.e., the higher the result, the smaller the extent of problems in this area.

In addition, the following relationship was found: the more night shifts per month, the worse the results in the assessment of functioning in the studied areas (Table 4). Kruskal Wallis tests were used. A statistically significantly higher rate in all domains and the entire FSFI questionnaire occurred in women who were not on night shifts.

Table 4 Female Sexual Function Index (PL-FSFI) results. Impact of the number of night shifts on the satisfaction with sex life.

A statistically significant difference in PL-FSFI results was found between the respondents using and not using contraception (Student’s *t*-test) in correlation with the data on the reproductive health of the examined women: the respondents using contraception achieved higher results in all domains. However, the type of work performed did not affect the use of contraception. The Chi-square test was used; the analysis did not show a relationship between the variables X2(1) = 0.50; *p* = 0.479.

In terms of having children, a relationship was demonstrated between the variables regarding the type of work performed X2(1) = 30.73; *p* = 0.000. Women who do not work shifts were more likely to have children (actual value > expected value). Subjects who had children (Mann–Whitney U test) achieved statistically significant higher results in the following domains: arousal Z = −2.29; *p* < 0.05, level of desire Z = −1.94; *p* = 0.05, confidence Z = −2.79; *p* < 0.05, lubrication Z = −2.90; *p* < 0.05, orgasm Z = −3.37; *p* = 0.001, frequency of orgasms Z = −2.41; *p* < 0.05, satisfaction with orgasms Z = −2.75; *p* < 0.05 , general sex life Z = −2.10; *p* < 0.05, pain following vaginal penetration Z = −1.90; *p* < 0.05.

Low negative correlations (Spearman’s correlation) were noted between the number of miscarriages and satisfaction in particular aspects of sexual life. The analysis showed that with the increase in the number of miscarriages, the level of satisfaction decreases (in the domains: satisfaction rHO = −0.30; *p* < 0.05, lubrication rHO = −0.29; *p* < 0.05, satisfaction with orgasm rHO = −0.27; *p* < 0.05, satisfaction rHO = −0.25; *p* < 0.05, pain during vaginal penetration rHO = −0.30; *p* < 0.05 and in the overall result rHO = −0.23; *p* < 0.05. The occurrence of miscarriages was statistically significantly more frequent in the group of women who worked in a shift system (X2 (2) = 15.47; *p* = 0.000). Declared reproductive problems (Table 2) occurred more frequently in women working shifts (X2(1) = 11.56; *p* = 0.001).

## 4. Discussion

Health systems require the provision of round-the-clock services for patient safety. Medical professionals should be aware that shift work may involve specific health risks. Disturbed circadian rhythm affects many areas of functioning and is associated with the risk of diseases [3,4,5,6,7,8,9,10]. Additionally, it was found that there may be a correlation between night work and reproductive health and sexual satisfaction [11,15,16,17,18,19].

Human and animal models clearly indicate that sleep deprivation affects the level of sex hormones, which are crucial in reproduction. It also affects the woman’s body: reproductive abilities depend on daily changes occurring in the circadian rhythm. Shift-induced circadian rhythm disorders affect reproductive health through the deregulation of sex steroid, gonadotropin, and prolactin production, which may result in infertility and abnormalities in the menstrual cycles of women working shifts. Sleep disturbances in shift workers cause reduced melatonin production and excessive activation of the hypothalamic–pituitary–adrenal (HPA) axis, resulting in early loss of pregnancies, failed embryo implantation, or lack of ovulation. Lack of sleep in women also affects the secretion of gonadotropins and sex steroids, which leads to infertility in women [19,23,24,25,26,27]. Our research has shown that reproductive problems, such as the failure to get pregnant for more than six months, miscarriage (also recurrent), and congenital fetal anomalies occurred in 23.7% of midwives working nights and only in 7.6% of midwives working days only.

So far, shift work including nights has been shown to negatively impact women’s sexual function and reproductive health, such as sexual desire, arousal, lubrication, orgasm, satisfaction, and pain during vaginal penetration [28,29]. Our results confirm that 51.4% of midwives working in the shift system have sexual disorders compared to 7.7% of midwives working during the day without night shifts. Sexual disorders are observed in the domains of desire, arousal, lubrication, orgasm, satisfaction, and pain. The larger the number of night shifts per month, the greater the severity of these disorders. Among shift workers lower scores in variables, which can have a bidirectional cause—result correlation has been revealed. Shift workers have fewer children, more miscarriages, and declared lower scores in sexual functioning and satisfaction. Both reproductive disruption and sexual dysfunction may play a role here. The results of this study are in line with disturbing reports concerning the professional situation of Polish nurses and midwives. The life expectancy is 61.4 years in this occupational group in the last five years, while the average life expectancy for women in Poland is 81.8 years [2]. Excessive workload, lower life expectancy than the general population of Polish women, and, apparently, significantly higher incidence of reproductive and sexual health disorders show the scale of problems that affect Polish midwives, especially those working shifts. That calls for strategies for reproductive and sexual health education and minimizing the impact of shift work on the health of midwives and other female professionals working shifts.

### Limitations

A small number of studies on the impact of shift work on midwives’ reproductive and sexual health can be found in the available literature, which significantly limits the possibility of comparing the obtained results. It is necessary to study larger groups of midwives and consider other variables, which may also show significant relationships.

The previous shift work experience length was included as an item in the research questionnaire. The statistical analysis included the current declared work system.

## 5. Conclusions

Aside from the results of this research, there are very few reports in Poland and the world on the impact of shift work on the reproductive and sexual health of the professional group of midwives. This is particularly interesting in the context of midwives’ professional duties, which include monitoring and diagnosing their patients’ reproductive and sexual health parameters. Furthermore, through their work, they are exposed to an increased risk of such problems. The presented research results indicate that in the group of midwives working shifts including nights, sexual health disorders and reproductive health disorders occur on a large scale more often than in the group working only during the day. The scale of occurrence of irregularities is alarming. It seems necessary to intensify preventive efforts among midwives, e.g., through additional research in occupational medicine units.

## Figures and Tables

**Table 1:** Characteristics of the group/general information about the respondents.

Characteristics	*N*	*%*
**Age**		
22–30	175	33.7
31–36	99	19
37–45	101	19.4
>46	145	27.9
**Place of residence**		
Rural	64	12.3
Urban	456	87.7
**Education**		
High school (medical)	55	10.6
Bachelor in Midwifery	227	43.7
Master of Science in Midwifery	237	45.6
PhD in Health Sciences	1	0.2
**Civil status**		
Single	77	14.8
Married	321	61.7
Cohabitating	81	15.6
Divorced	24	4.6
Widow	10	1.9
Separated	7	1.3
**Work experience**		
6 months–5 years	161	31.0
5–10 years	96	18.5
11–15 years	57	11.0
over 16 years	206	39.6
**Shift work experience**		
less than 5 years	139	26.7
5–10 years	100	19.2
11–15 years	30	5.8
over 16 years	147	28.3
no experience in shift work	104	20.0
**Average number of night shifts during the month**		
0	107	20.6
1–3	16	3.1
4–6	190	36.5
7–9	180	34.6
10 and more	27	5.2

**Table 2:** Reproductive health of midwives working night shifts and working days only.

Characteristics	Shift Work with Night Shifts		Working Days Only without Night Shifts	
	N *	*%*	N *	*%*
	416	100	104	100
**Cycle regularity**				
Regular	298	75.3	74	74.7
Irregular	98	24.7	25	25.3
**Length of the menstrual cycle**				
20–25 days	23	5.9	10	11.8
26–30 days	255	64.9	69	81.2
31–35 days	84	21.4	5	5.9
more than 35 days	31	7.9	1	1.2
**Children**				
Yes	239	58.9	52	50.0
No	158	38.9	45	43.3
No, but I am trying.	9	2.2	7	6.7
**Number of pregnancies**				
0	101	28.9	8	8.6
1	70	20.1	13	14.0
2	117	33.5	54	58.1
3	30	8.6	15	16.1
4	26	7.4	0	0
5	4	1.1	3	3.2
6	1	0.3	0	0
**Number of miscarriages**				
0	262	80.1	86	95.6
1	50	15.3	2	2.2
2	13	4.0	0	0
3	2	0.6	2	2.2
**Reproductive problems**				
Failure to become pregnant within more than 6 months of trying	33	7.9	1	1.0
Miscarriage in the first trimester of pregnancy	46	11.1	4	3.8
Fetal congenital anomalies	1	0.2	0	0
Recurrent spontaneous miscarriages (3 or more)	2	0.5	0	0
Others	9	2.2	0	0
**Contraception used**				
Condom	76	18.3	18	17.3
Oral contraceptive pill	77	18.5	14	13.5
IUD	47	11.3	17	16.3
Vaginal ring	4	1	4	3.8
Birth control patches	2	0.5	1	1
Implants	3	0.7	0	0
Hormonal Injection	0	0	1	1
None	207	49.8	49	47.1

* *N* numbers vary due to missing data.

**Table 3:** Female Sexual Function Index (PL-FSFI) results.

EN-FSFI Domains:	Shift Work with Night Shifts (*N* = 416)				Working Days Only without Night Shifts (*N* = 104)			
	Min	Max	M	SD	Min	Max	M	SD
DESIRE	0	6	3.60	1.33	0	6	4.95	1.36
AROUSAL	0	6	3.80	1.76	0	6	5.07	1.54
LUBRICATION	0	6	4.38	1.88	0	6	5.42	1.59
ORGASM	0	6	4.04	1.92	0	6	5.34	1.60
SATISFACTION	0	6	4.32	1.70	0	6	5.41	1.38
PAIN	0	6	4.70	2.05	0	6	5.29	1.68
OVERALL SCORE	0	36	24.86	9.32	0	36	31.51	8.71

Statistics presented: mean_standard deviation or *n* (%).

**Table 4:** The impact of the number of night shifts on sex life satisfaction.

Domains and Questions	Number of Night Shifts Per Month										Kruskal-Wallis (H)
	0		1–3		4–6		7–9		10 i>		
	M	SD	M	SD	M	SD	M	SD	M	SD	
**DESIRE**	5.05	1.17	3.63	1.52	3.6	1.35	3.61	1.3	3	1.44	106.22; *p* = 0.000
1. Frequency	4.23	1.04	2.88	1.5	3.03	1.22	2.97	1.24	2.71	1.2	83.72; *p* = 0.000
2.Level of desire	4.32	0.78	3.19	1.33	3.13	1.02	3.11	1.02	2.92	0.83	112.05; *p* = 0.000
**AROUSAL**	5.17	1.36	3.24	2.34	3.77	1.82	3.92	1.64	3.18	1.95	88.23; *p* = 0.000
3.Frequency	4.46	1.11	3.19	2.04	3.37	1.65	3.4	1.53	3.21	1.61	50.14; *p* = 0.000
4. Level of desire	4.2	0.99	2.81	2.32	3.16	1.4	3.22	1.25	2.92	1.32	74.29; *p* = 0.000
5.Confidence	4.27	1.04	2.56	2.13	3.15	1.41	3.28	1.32	2.96	1.27	75.05; *p* = 0.000
6.Satisfaction	4.63	1.07	2.25	2.24	3.23	1.82	3.34	1.7	2.83	1.88	75.18; *p* = 0.000
**LUBRICATION**	5.54	1.38	3.08	2.86	4.29	2	4.58	1.61	3.91	2.18	85.53; *p* = 0.000
7.Frequency	4.74	0.98	2.5	2.39	3.66	1.74	3.82	1.54	3.33	1.88	57.89; *p* = 0.000
8.Difficulty	4.74	0.98	2.69	2.47	3.78	1.57	3.84	1.36	3.88	1.51	78.31; *p* = 0.000
9.Frequency maintaining	4.74	0.98	2.44	2.31	3.55	1.74	3.8	1.56	3.88	1.68	64.85; *p* = 0.000
10.Difficulty maintaining	4.6	1.9	2.63	2.42	3.74	1.59	3.98	1.31	3.58	1.69	53.80; *p* = 0.000
**ORGASM**	5.47	1.4	2.98	2.75	3.93	2.01	4.23	1.68	3.53	2.22	89.91; *p* = 0.000
11.Frequency	4.6	1.07	2.5	2.34	3.39	1.75	3.54	1.57	3.17	1.88	57.83; *p* = 0.000
12.Difficulty	4.7	0.99	2.44	2.25	3.45	1.58	3.65	1.35	3.33	1.76	99.00; *p* = 0.000
13.Satisfaction	4.63	1	2.5	2.31	3.25	1.73	3.51	1.51	3.42	1.59	77.73; *p* = 0.000
**SATISFACTION**	5.52	1.21	3.6	2.46	4.21	1.78	4.5	1.5	3.78	1.96	76.91; *p* = 0.000
14. with closeness	4.66	1	2.69	2.47	3.61	1.66	3.94	1.43	3.38	1.64	53.00; *p* = 0.000
15. with sexual relationship	4.82	0.39	4.08	1.38	3.76	1.34	3.78	1.26	3.75	1.19	71.01; *p* = 0.000
16. with overall sexual life	4.77	0.55	3.69	1.44	3.6	1.32	3.75	1.26	3.5	1.29	79.64; *p* = 0.000
**PAIN**	5.43	1.5	3.83	2.76	4.59	2.17	4.9	1.82	4.15	2.46	14.99; *p* = 0.000
17.During vaginal penetration	4.72	1.02	3.64	2.02	3.99	1.71	4.16	1.57	3.79	1.82	26.15; *p* = 0.000
18.After vaginal penetration	4.69	1.02	3.57	2.21	4.04	1.71	4.25	1.53	3.96	1.83	14.52; *p* = 0.000
19.Level of pain during or after vaginal penetration	4.55	1.03	3.25	2.32	3.88	1.72	4.05	1.47	3.92	1.61	12.57; *p* = 0.000
**OVERALL SCORE**	32.18	7.37	20.36	13.23	24.4	9.84	25.75	8.18	21.54	11.35	120.39_;_ *p* = 0.000

M—median. SD—standard deviation.

## Data Availability

Data available on request due to privacy/ethical restrictions.
